# Supplementary material for: The interplay between insomnia symptoms and Alzheimer’s disease across three main brain networks
Source: Sleep. 2024 Jun 27;47(10):zsae145. doi: 10.1093/sleep/zsae145 (PMC11467060; doi:10.1093/sleep/zsae145)
Supplement: zsae145_suppl_Supplementary_Materials [file zsae145_suppl_supplementary_materials.docx]

## The interplay between insomnia symptoms and Alzheimer’s Disease across three main brain networks: supplementary materials.

Jorik D. Elberse^1,2,3^, Amin Saberi^1,2,4^, Reihaneh Ahmadi^1,5^, Monir Changizi^6^, Hanwen Bi^1,2^, Felix Hoffstaedter^1,2^, Bryce A. Mander^7^, Simon B. Eickhoff^1,2^, Masoud Tahmasian^1,2,8^*, for the Alzheimer's Disease Neuroimaging Initiative**

1. Institute of Neuroscience and Medicine, Brain and Behavior (INM-7), Research Center Jülich, Jülich, Germany
2. Institute of Systems Neuroscience, Medical Faculty and University Hospital Düsseldorf, Heinrich Heine University, Düsseldorf, Germany
3. Max Planck School of Cognition, Leipzig, Germany
4. Otto Hahn Group Cognitive Neurogenetics, Max Planck Institute for Human Cognitive and Brain Sciences, Leipzig, Germany
5. Faculty of Medicine, Julius-Maximilians University of Würzburg, Würzburg, Germany
6. Department of Neurological Diseases, Shahid Beheshti University of Medical Sciences, Tehran, Iran
7. Department of Psychiatry and Human Behavior, University of California, Irvine, CA 92603, USA
8. Department of Nuclear Medicine, University Hospital and Medical Faculty, University of Cologne, Cologne, Germany

***Corresponding Author:** Masoud Tahmasian, Institute of Neuroscience and Medicine, Brain & Behaviour (INM-7), Research Centre Jülich, Wilhelm-Johnen-Straße,52428 Jülich, Germany. Telephone: +49 2461 61-8785, Fax: +49 2461 61-1880. Email: [m.tahmasian@fz-juelich.de](mailto:m.tahmasian@fz-juelich.de)

****Data Usage**: Data used in preparation of this article were obtained from the Alzheimer’s Disease Neuroimaging Initiative (ADNI) database (adni.loni.usc.edu). As such, the investigators within the ADNI contributed to the design and implementation of ADNI and/or provided data but did not participate in the analysis or writing of this report. A complete listing of ADNI investigators can be found at: <http://adni.loni.usc.edu/wp-content/uploads/how_to_apply/ADNI_Acknowledgement_List.pdf>

|  | **FC** | **MMSE** | **GDS** | **NPI-C** | **NPI-D** | **NPI-E** | **NPI-G** | **NPI-H** | **NPI-I** | **NPI-K** | **NPI-L** |
| --- | --- | --- | --- | --- | --- | --- | --- | --- | --- | --- | --- |
| **(A) Cognitively Normal (CN)** | | | | | | | | | | | |
| *(1) lDMN_PFC − lDMN_pCunPCC* | 0.04**** | -0.3944** | -0.4508** | 0.0818* |  |  |  | -0.0211* |  | 0.8883**** | 0.0599* |
| *(2) lDMN_PFC − rDMN_pCunPCC* | 0.03*** | -0.2755* | -0.5731**** | 0.0844* |  | 0.0409* |  |  |  | 0.9643**** | 0.0766* |
| *(3) lSN_FrOperIns − rSN_Med* | -0.05** |  |  | 0.1765* |  |  |  |  |  |  | 0.1849** |
| *(4) rSN_TempOccPar − rCEN_PFCl* | 0.03* |  | -0.4974** |  |  |  |  |  |  | 0.6936* |  |
| *(5) rSN_Med − rCEN_PFCl* | 0.03* |  | -0.503** |  | 0.3755** |  |  |  |  | 0.7673** |  |
| *(6) rSN_Med − lDMN_pCunPCC* | -0.05* |  |  |  |  |  |  |  |  |  |  |
| *(7) rDMN_Temp − rDMN_pCunPCC* | -0.03* |  | -0.9121**** |  |  |  |  |  |  |  | 0.1059* |
| *(8) rDMN_Par − rCEN_PFCl* | 0.04* |  | -1.0677*** |  |  |  |  |  |  | 1.3761** |  |
| *(9) rCEN_PFCl − rCEN_PFCl* | 0.02* |  | -0.7284* | 0.2538*** |  |  |  |  |  | 1.0444* |  |
| *(10) rCEN_Par − rCEN_PFCl* | 0.03* |  | -0.4691* |  |  |  |  |  |  | 0.8362** | 0.1109** |
| *(11) lSN_FrOperIns − lSN_Med* | -0.03* |  | -0.5562* | 0.1568** |  |  | 0.1488* |  |  |  | 0.1409** |
| *(12) lDMN_Temp − lDMN_PFC* | 0.02* | -0.3227* | -1.0721**** |  |  | 0.0469* |  |  | -0.1504* |  |  |
| *(13) lDMN_PFC − lCEN_PFCl* | 0.03* |  | -0.3987* | 0.1549** |  | 0.1002*** | -0.148* |  | 0.2069* |  |  |
| *(14) lDMN_Par − lDMN_PFC* | 0.03* | -0.2529* | -0.4554*** |  |  | 0.0592*** |  |  |  | 0.4369* |  |
| **(B) Mild Cognitive Impairment (MCI)** | | | | | | | | | | | |
| *(1) rDMN_PFCdPFCm − rDMN_pCunPCC* | 0.04** | -0.7019* | -0.6852* |  | -0.3899* |  | -0.4664* |  |  |  | -0.1096* |
| *(2) lDMN_PFC − lDMN_pCunPCC* | 0.03** | -0.5631** | -1.2982**** |  | -0.4081**** |  |  |  |  | 0.6345** |  |
| *(3) lDMN_pCunPCC − rDMN_PFCdPFCm* | 0.04** | -0.9061** | -0.9307** |  | -0.417* |  |  |  |  | 0.9367* |  |
| *(4) rSN_TempOccPar − rSN_Med* | 0.05* |  | -0.9242* |  |  |  |  |  |  | 1.9186**** |  |
| *(5) rSN_TempOccPar - lDMN_pCunPCC* | -0.05* |  |  |  |  |  |  | 0.1988**** |  |  | 0.1183* |
| *(6) rSN_FrOperIns − rSN_Med* | 0.06* |  | -1.7228** |  |  |  |  |  |  | 1.9327* |  |
| *(7) rDMN_PFCdPFCm − rCEN_PFCl* | -0.03* |  | -0.6662*** |  | 0.2709** |  |  | 0.0789* |  |  |  |
| *(8) rDMN_PFCdPFCm − lCEN_pCun* | 0.05* | -1.1715* |  |  |  |  |  |  |  |  |  |
| *(9) lSN_Med − rSN_TempOccPar* | 0.04* |  | -1.3482**** |  |  |  |  |  | -0.4702* | 1.5414**** |  |
| *(10) lSN_Med − rDMN_Temp* | 0.03* |  | -1.1583**** |  | 0.2957* |  |  | 0.0909* | -0.3378* | 1.175**** |  |
| *(11) lSN_FrOperIns − rSN_Med* | 0.04* |  | -0.9356* |  |  |  |  |  |  | 1.5935** |  |
| *(12) lDMN_Temp − rDMN_PFCdPFCm* | 0.04* |  | -1.9384**** |  |  |  |  |  |  | 0.8637* | -0.1199* |
| *(13) lDMN_PFC − rDMN_pCunPCC* | 0.02* | -0.5408** | -0.9398**** |  | -0.3273** |  | -0.2688* |  | -0.3112* | 0.564* |  |
| **(C) Alzheimer's Disease (AD)** | | | | | | | | | | | |
| *(1) rDMN_PFCv − rCEN_Par* | -0.13**** |  | -2.0113** |  |  |  | 2.4343* |  |  | -4.0445*** |  |
| *(2) lDMN_PFC − rCEN_Par* | -0.12**** |  | -1.6419**** |  |  |  |  |  |  | -2.0621**** |  |
| *(3) lSN_Med − rCEN_PFCl* | -0.07*** | -2.1171* | -0.7098* |  |  |  |  | 0.2123** | 0.6052* |  |  |
| *(4) rSN_TempOccPar − rDMN_pCunPCC* | -0.12** |  |  |  |  |  | 1.7466* |  |  |  |  |
| *(5) rSN_Med − rCEN_PFCl* | -0.08** |  |  |  |  |  |  | 0.2037* |  |  |  |
| *(6) rDMN_PFCv − rDMN_pCunPCC* | -0.11** |  | -1.3818* | 1.0339* |  |  |  |  | 1.08* |  | 2.4807* |
| *(7) lSN_Med − rCEN_Par* | -0.09** |  | -0.9273* |  |  |  | 2.051* | 0.3286** |  |  |  |
| *(8) lSN_FrOperIns − rDMN_pCunPCC* | -0.13** |  |  |  |  |  |  |  |  |  |  |
| *(9) lSN_FrOperIns − rCEN_Par* | -0.11** | 3.9084* |  |  |  |  |  |  |  |  |  |
| *(10) lDMN_Temp − rDMN_Temp* | -0.09** |  |  |  |  |  |  |  |  |  |  |
| *(11) lDMN_PFC − rCEN_PFCmp* | -0.06** |  |  |  | 0.9438* | 0.7574* |  |  | 1.0424* |  |  |
| *(12) lDMN_PFC − rCEN_PFCl* | -0.03** |  |  |  | 0.3583* | 0.6007**** | 0.8617* |  |  | 0.6773* |  |
| *(13) lDMN_Par − rDMN_Temp* | -0.08** | 3.7662** |  |  |  |  | 2.2371** | -0.2405* | -0.8378* |  |  |
| *(14) lCEN_Par − rCEN_PFCl* | -0.1** |  |  |  |  |  |  |  |  |  | 2.7278* |
| *(15) rSN_TempOccPar − rDMN_Temp* | -0.08* |  |  |  |  |  | 1.5987* |  |  |  |  |
| *(16) rSN_TempOccPar − lDMN_Temp* | -0.12* |  |  |  |  |  |  |  |  |  |  |
| *(17) rSN_TempOccPar − lDMN_PFC* | -0.05* | 2.0374* | -1.3407**** | 0.5001* |  |  | 1.5194** |  |  |  |  |
| *(18) rSN_Med − rCEN_Par* | -0.09* |  |  |  |  |  |  | 0.2526* |  |  |  |
| *(19) rSN_FrOperIns − rDMN_pCunPCC* | -0.14* |  |  |  |  |  |  |  |  |  |  |
| *(20) rDMN_Temp − rDMN_Temp* | -0.08* |  |  |  |  |  |  |  |  |  |  |
| *(21) rDMN_PFCv − rCEN_pCun* | -0.12* |  | -1.9915* |  |  |  |  |  |  |  |  |
| *(22) rDMN_PFCv − lCEN_pCun* | -0.12* |  | -1.8595* |  |  |  | 3.9403* |  |  |  |  |
| *(23) rDMN_PFCv − lCEN_Par* | -0.1* |  |  |  |  |  |  |  |  |  |  |
| *(24) rDMN_pCunPCC − rCEN_PFCl* | -0.05* |  | -1.7767**** |  |  |  |  |  |  |  | 1.5073* |
| *(25) rDMN_pCunPCC − rCEN_Par* | -0.11* |  |  |  |  |  |  |  |  |  |  |
| *(26) rDMN_pCunPCC − lCEN_Par* | -0.12* |  |  |  |  |  |  |  |  |  |  |
| *(27) rDMN_Par − rDMN_PFCv* | -0.11* |  |  |  |  |  |  |  |  |  |  |
| *(28) rDMN_Par − rDMN_pCunPCC* | -0.13* |  |  |  |  |  |  |  |  |  |  |
| *(29) rDMN_Par − rCEN_PFCl* | 0.07* |  | -1.4755* |  |  |  |  |  |  |  | 2.5697* |
| *(30) rCEN_Par − rCEN_pCun* | -0.11* |  |  |  |  |  |  |  |  |  |  |
| *(31) rCEN_Par − rCEN_Cing* | -0.11* |  |  |  |  |  |  |  |  |  |  |
| *(32) lSN_Med − rDMN_PFCdPFCm* | -0.06* |  |  |  |  |  |  |  |  |  |  |
| *(33) lSN_Med − lDMN_PFC* | -0.03* |  | -0.5632* |  |  |  |  |  | 0.6133** |  |  |
| *(34) lSN_FrOperIns − lDMN_pCunPCC* | -0.1* |  |  |  |  |  | 2.0375* |  |  |  |  |
| *(35) lDMN_Temp − rDMN_pCunPCC* | -0.1* |  |  |  |  |  |  | -0.3172* |  |  |  |
| *(36) lDMN_PFC − rCEN_Cing* | -0.06* |  | -1.6888*** |  |  |  |  |  |  |  |  |
| *(37) lDMN_pCunPCC − rDMN_Par* | -0.12* |  |  |  |  |  |  |  |  |  |  |
| *(38) lDMN_Par − rDMN_PFCv* | -0.09* |  |  |  |  |  |  |  |  |  | 2.8594* |
| *(39) lCEN_pCun − rCEN_Par* | -0.13* |  |  |  |  |  | 2.9559* |  |  |  |  |
| *(40) lCEN_Cing − rCEN_Par* | -0.14* |  |  |  |  |  | 3.3607* |  |  |  |  |

**Table S1 – Post-Hoc Correlations between FC Alterations and Cognitive and Affective Scores.** Edges with insomnia-related FC alterations in the (**A**) Cognitively Normal (**B**) Mild Cognitive Impairment, and (**C**) Alzheimer’s Disease groups, listed in order from most to least significant, alongside correlations with MMSE, GDS, NPI-C (Aggression/Agitation), NPI-D (Depression/Dysphoria), NPI-E (Anxiety), NPI-G (Apathy), NPI-H (Disinhibition), NPI-I (Irritability), NPI-K (Sleep), and NPI-L (Appetite) scores. *CEN = Central Executive Network; Cing = cingulate; DMN = Default Mode Network; Fr = frontal; GDS = Geriatric Depression Scale; Ins = insula; Med = medial; MMSE = Mini-Mental State Exam; NPI = Neuropsychiatric Index; Occ = occipital; Oper = operculum; Par = parietal; PCC = posterior cingulate cortex; pCun = precuneus; PFC = prefrontal cortex; PFCd = dorsal prefrontal cortex; PFCl = lateral prefrontal cortex; PFCm = medial prefrontal cortex; PFCmp = medial posterior prefrontal cortex; PFCv = ventral prefrontal cortex; SN = Salience Network; Temp = temporal. *p < 0.05, **p < 0.01, ***p < 0.001, ****p < 0.0001, FDR corrected.*


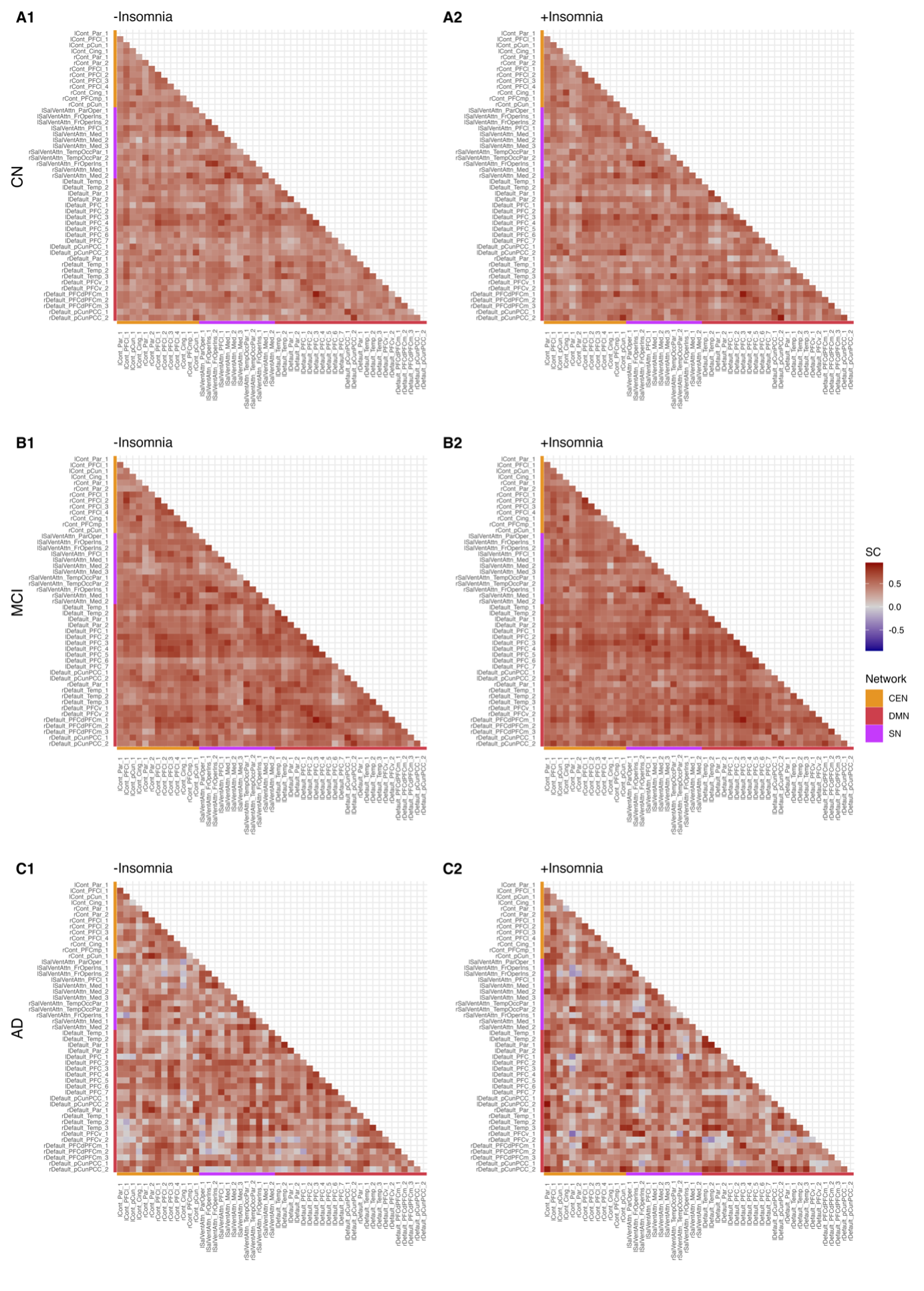


**Figure S1 – Three Network Structural Covariance in the Presence of Insomnia Symptoms.** Node-level alterations in three network SC associated within(**A**) CN, (**B**) MCI, and (**C**) AD, and across the (**1**) -Insomnia and (**2**) +Insomnia conditions. A high SC was observed between nearly all nodes of the triple network. Permutation tests revealed no significant differences between -Insomnia and +Insomnia columns. *CEN = Central Executive Network; Cing = cingulate; DMN = Default Mode Network; Fr = frontal; Ins = insula; Med = medial; Occ = occipital; Oper = operculum; Par = parietal; PCC = posterior cingulate cortex; pCun = precuneus; PFC = prefrontal cortex; PFCd = dorsal prefrontal cortex; LPFC = lateral prefrontal cortex; PFCm = medial prefrontal cortex; PFCmp = medial posterior prefrontal cortex; PFCv = ventral prefrontal cortex; SC = Structural Covariance; SN = Salience Network; Temp = temporal.*


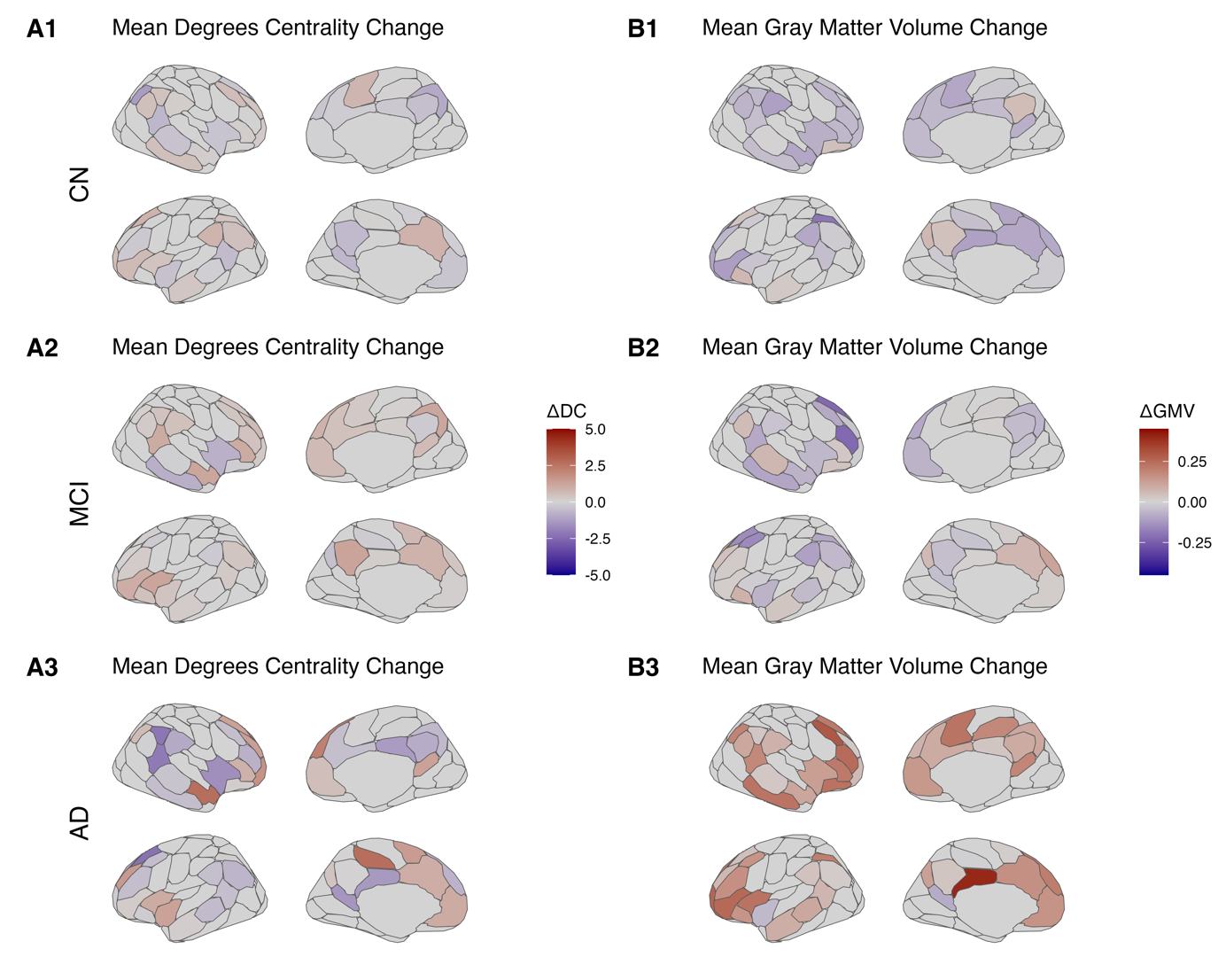


**Figure S2 – Mean Degrees Centrality and Gray Matter Volume Changes associated with Insomnia Symptoms.** Mean node-level (**A**) DC and (**B**) GMV changes associated with insomnia symptoms across (**1**) CN, (**2**) MCI, and (**3**) AD. Insomnia symptoms were associated with various patterns of non-significant DC and GMV changes within each diagnostic group. *∆DC = mean degrees centrality change; ∆GMV = mean gray matter volume change.*


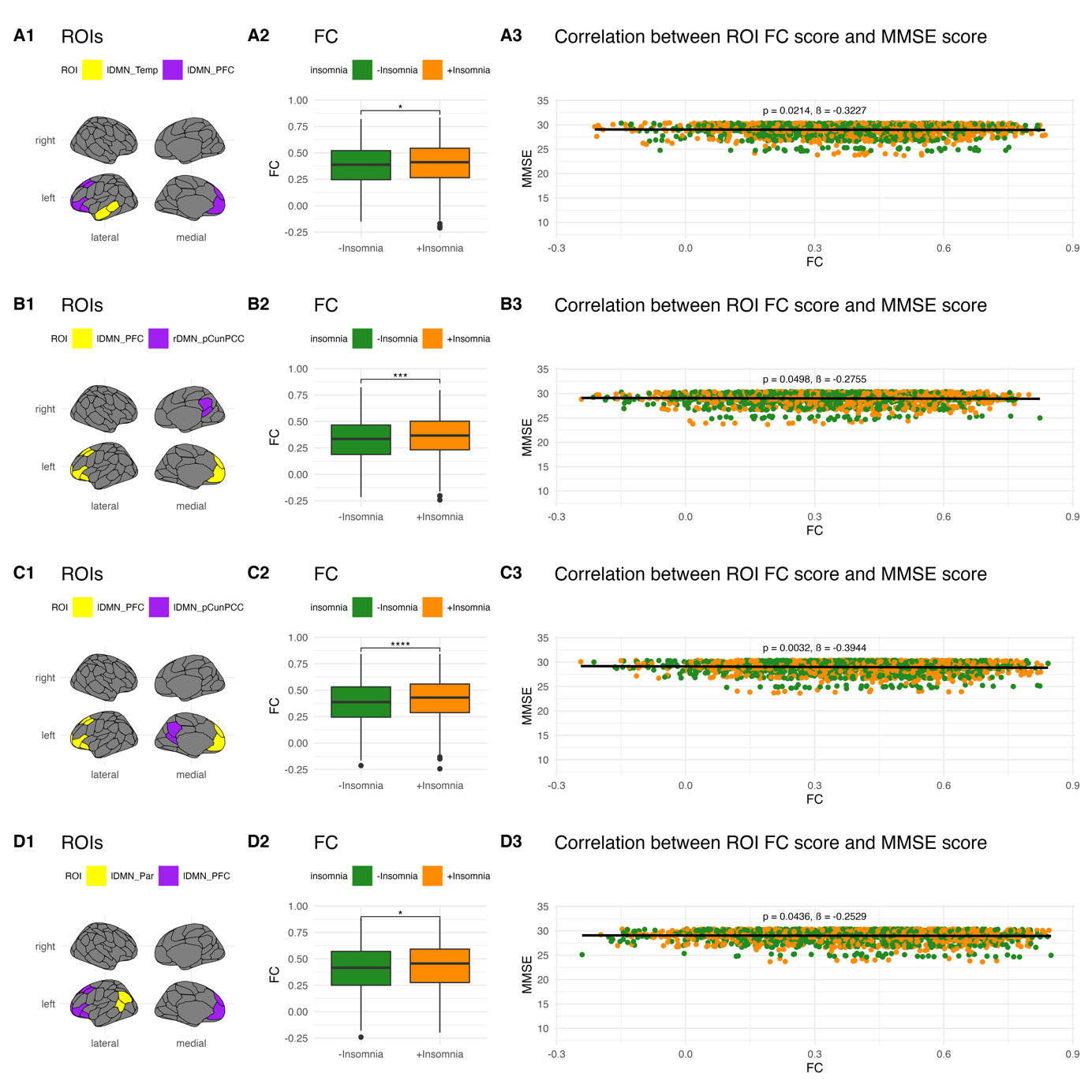


**Figure S3 - Abnormal Inter-ROI Connectivity and MMSE Score in CN.** ROI-level edges in CN +insomnia that predict significant changes in MMSE score. (**1**) ROI-level edges affected by the +insomnia condition. (**2**) Change in FCfor selected edges across the +insomnia condition. (**3**) Overall correlation of ROI-level edge FC scores and MMSE scores. Hyperconnective intra-DMN edges are overwhelmingly associated with decreased cognitive functioning in CN +insomnia. *CEN = Central Executive Network; DMN = Default Mode Network; FC = functional connectivity; Fr = frontal; Ins = insula; Med = medial; Occ = occipital; Oper = operculum; Par = parietal; PFC = prefrontal cortex; LPFC = lateral prefrontal cortex; SN = Salience Network; Temp = temporal. *p < 0.05, **p < 0.01, ***p < 0.001, ****p < 0.0001. ß = estimate.*


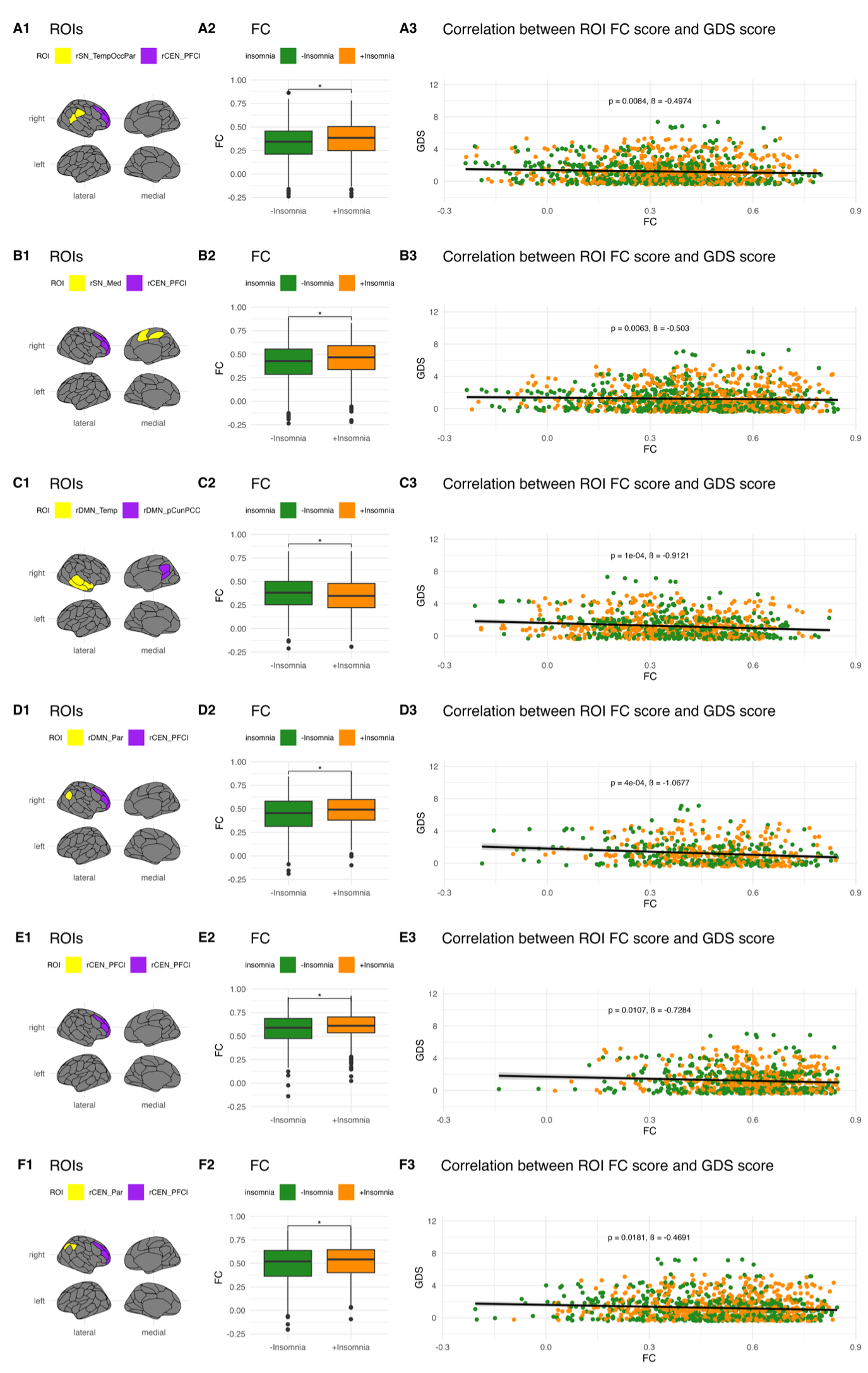


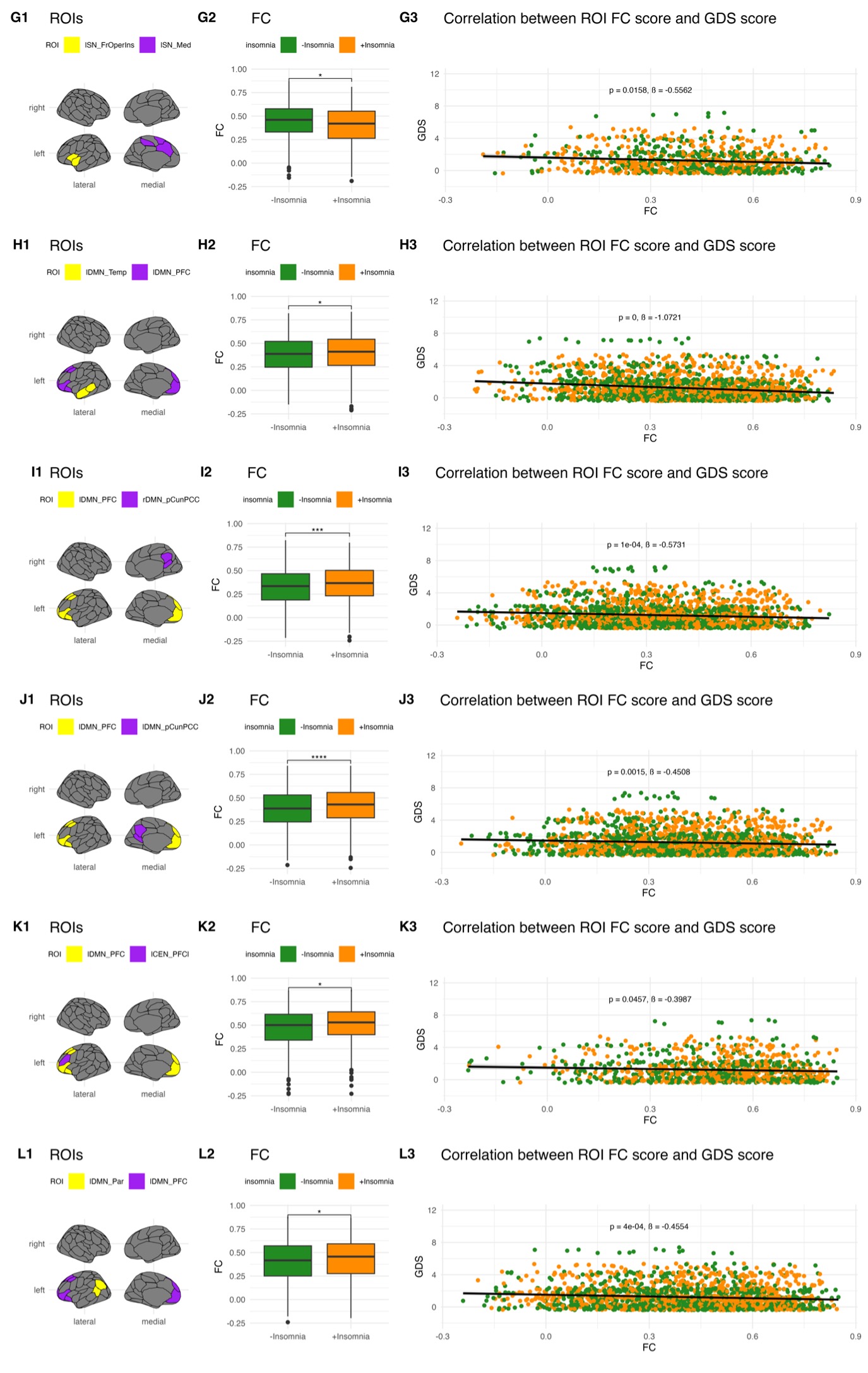


**Figure S4 - Abnormal Inter-ROI Connectivity and GDS Score in CN.** ROI-level edges in CN +insomnia that predict significant changes in GDS score. (**1**) ROI-level edges affected by the +insomnia condition. (**2**) Change in FCfor selected edges across the +insomnia condition. (**3**) Overall correlation of ROI-level edge FC scores and GDS scores. Hyperconnective intra-DMN edges are overwhelmingly associated with decreased cognitive functioning in CN +insomnia. *CEN = Central Executive Network; Cing = cingulate; DMN = Default Mode Network; FC = Functional Connectivity; Fr = frontal; GDS = Geriatric Depressio Score; Ins = insula; Med = medial; Occ = occipital; Oper = operculum; Par = parietal; PCC = posterior cingulate cortex; pCun = precuneus; PFC = prefrontal cortex; PFCd = dorsal prefrontal cortex; LPFC = lateral prefrontal cortex; PFCm = medial prefrontal cortex; PFCmp = medial posterior prefrontal cortex; PFCv = ventral prefrontal cortex; SN = Salience Network; Temp = temporal. *p < 0.05, **p < 0.01, ***p < 0.001, ****p < 0.0001. ß = estimate.*


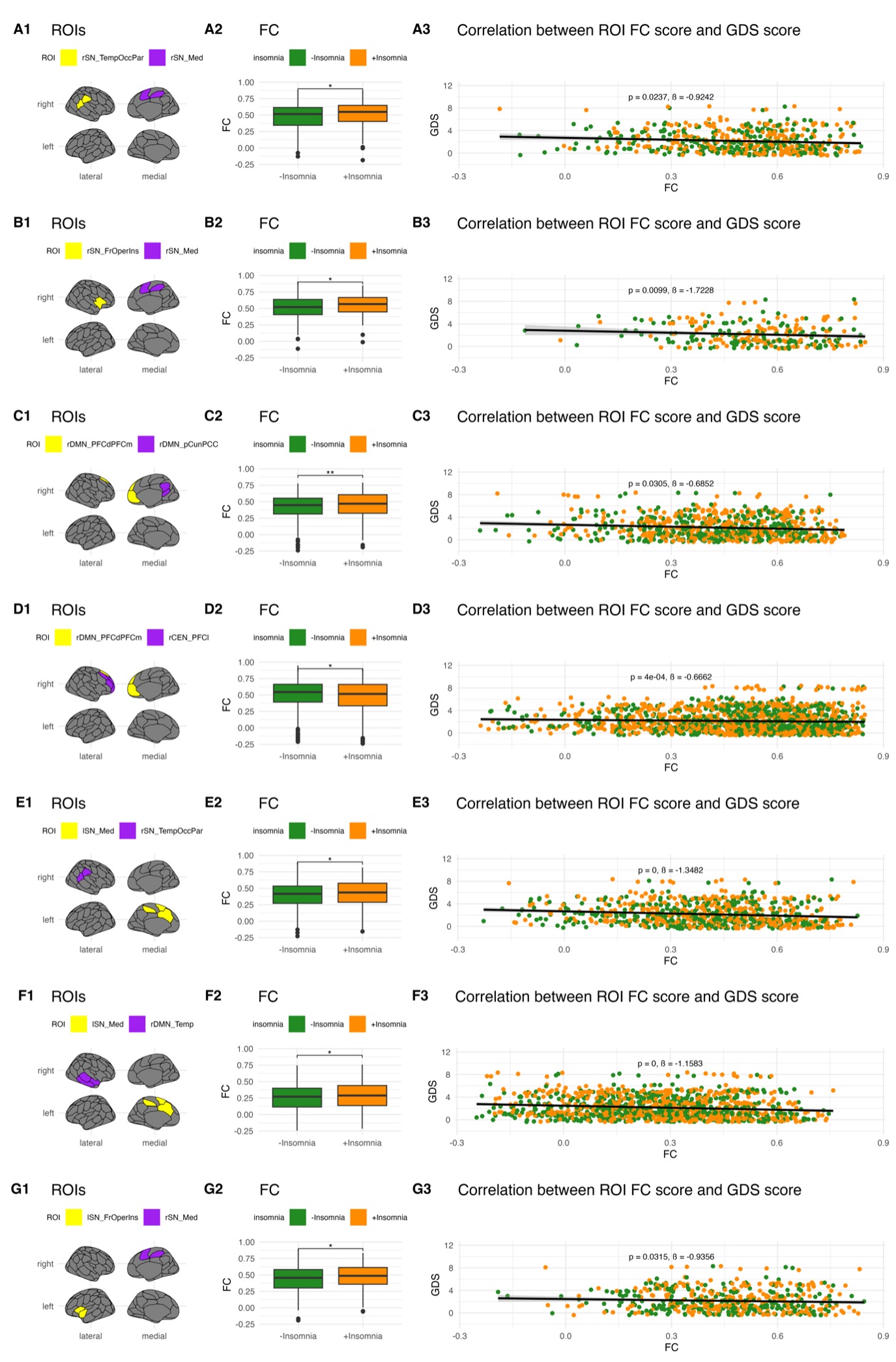


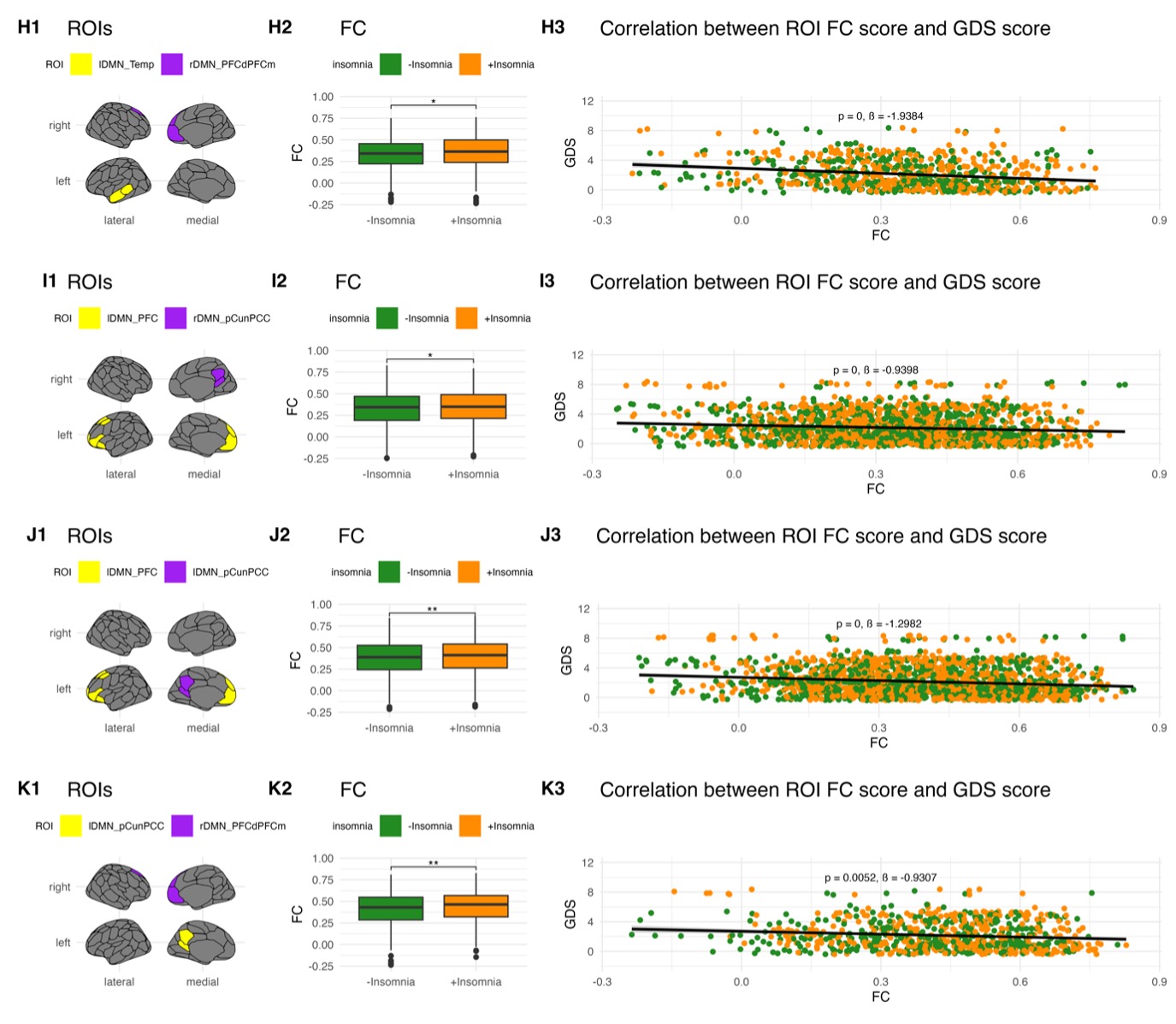


**Figure S5 - Abnormal Inter-ROI Connectivity and GDS Score in MCI.** ROI-level edges in MCI +insomnia that predict significant changes in GDS score. (**1**) ROI-level edges affected by the +insomnia condition. (**2**) Change in FCfor selected edges across the +insomnia condition. (**3**) Overall correlation of ROI-level edge FC scores and GDS scores. Hyperconnective intra-DMN edges are overwhelmingly associated with decreased cognitive functioning in MCI +insomnia. *CEN = Central Executive Network; Cing = cingulate; DMN = Default Mode Network; FC = Functional Connectivity; Fr = frontal; GDS = Geriatric Depressio Score; Ins = insula; Med = medial; Occ = occipital; Oper = operculum; Par = parietal; PCC = posterior cingulate cortex; pCun = precuneus; PFC = prefrontal cortex; PFCd = dorsal prefrontal cortex; LPFC = lateral prefrontal cortex; PFCm = medial prefrontal cortex; PFCmp = medial posterior prefrontal cortex; PFCv = ventral prefrontal cortex; SN = Salience Network; Temp = temporal. *p < 0.05, **p < 0.01, ***p < 0.001, ****p < 0.0001. ß = estimate.*


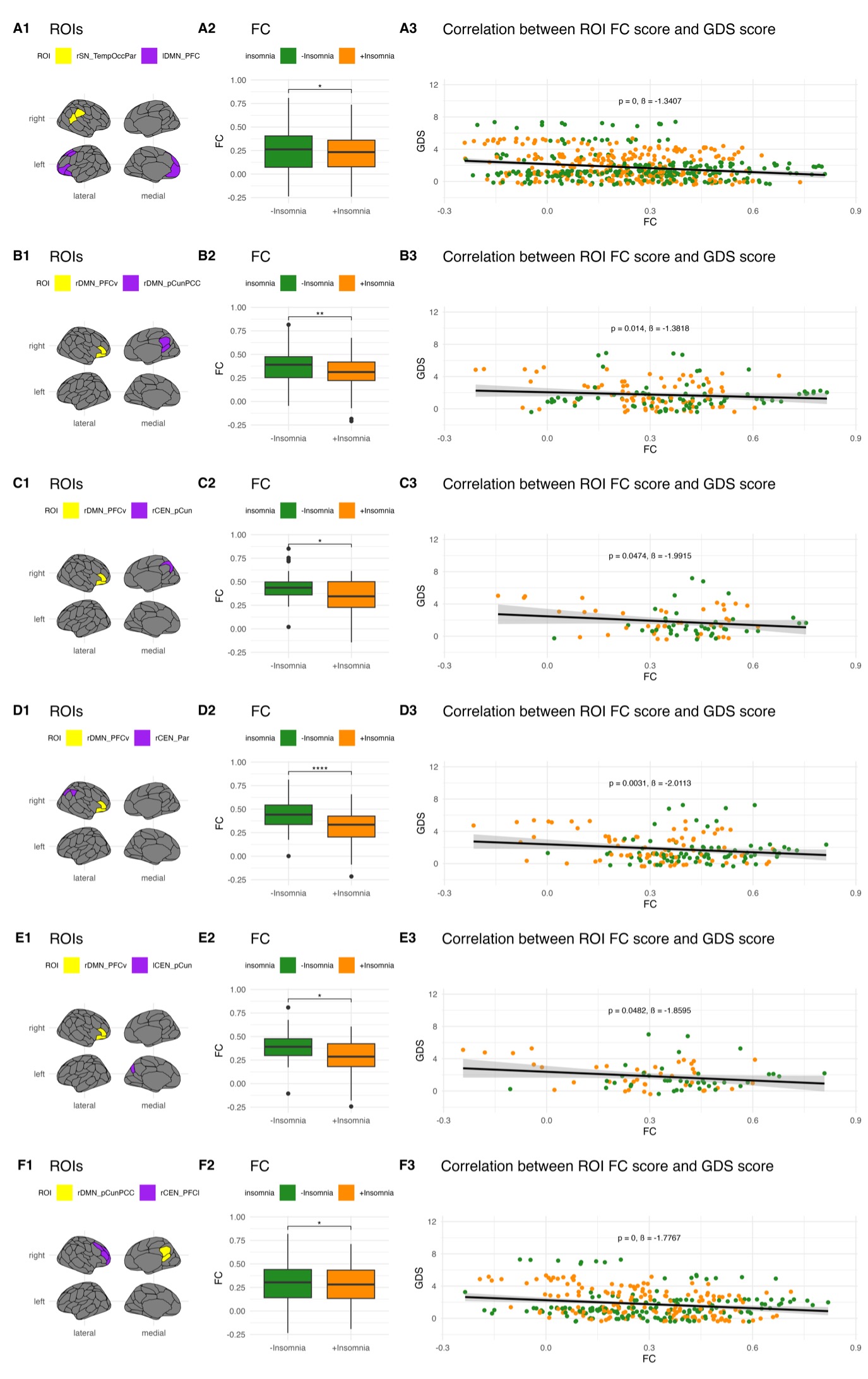

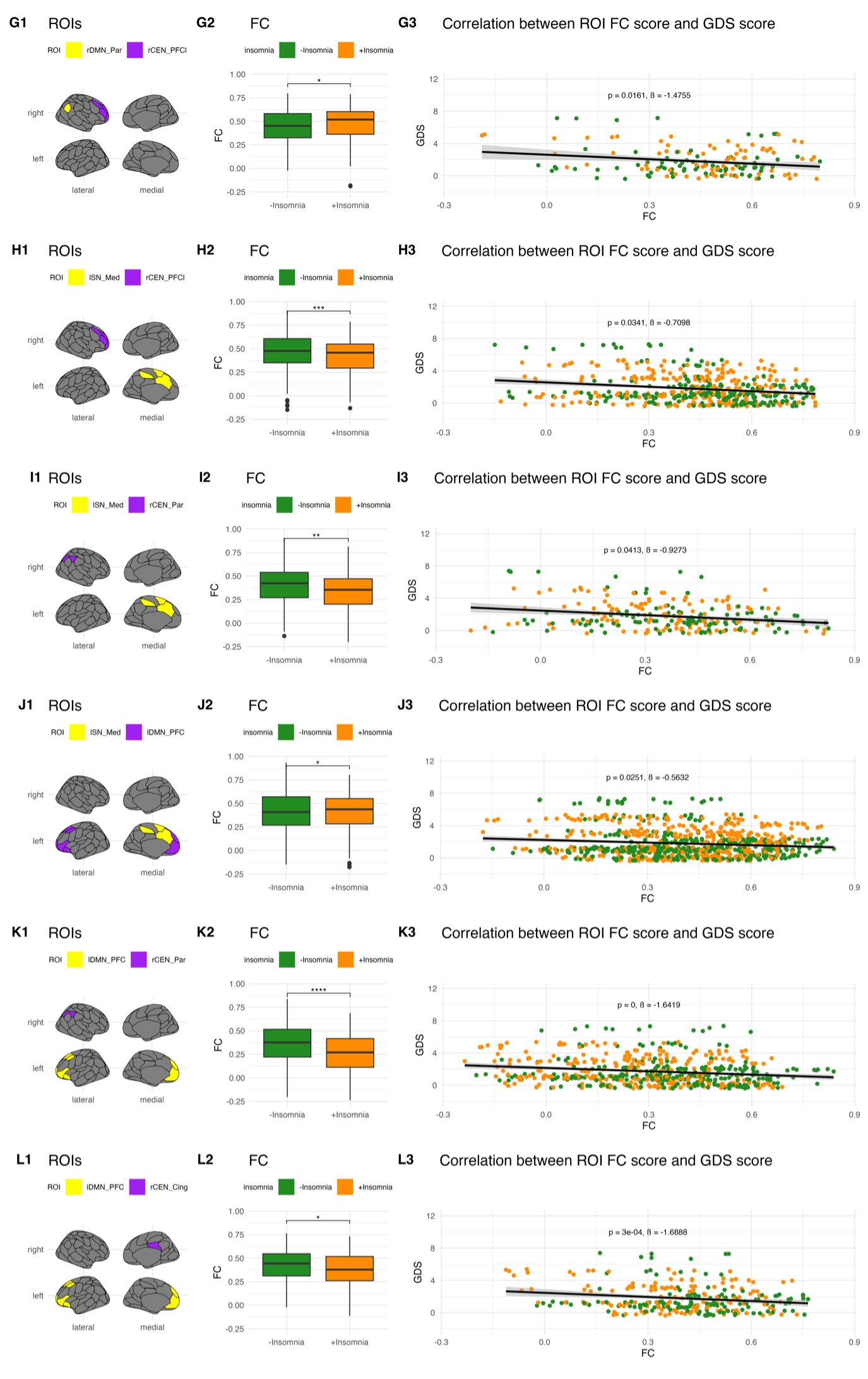


**Figure S6 - Abnormal Inter-ROI Connectivity and GDS Score in AD.** ROI-level edges in AD +insomnia that predict significant changes in GDS score. (**1**) ROI-level edges affected by the +insomnia condition. (**2**) Change in FCfor selected edges across the +insomnia condition. (**3**) Overall correlation of ROI-level edge FC scores and GDS scores. Hyperconnective intra-DMN edges are overwhelmingly associated with decreased cognitive functioning in AD +insomnia. *CEN = Central Executive Network; Cing = cingulate; DMN = Default Mode Network; FC = Functional Connectivity; Fr = frontal; GDS = Geriatric Depressio Score; Ins = insula; Med = medial; Occ = occipital; Oper = operculum; Par = parietal; PCC = posterior cingulate cortex; pCun = precuneus; PFC = prefrontal cortex; PFCd = dorsal prefrontal cortex; LPFC = lateral prefrontal cortex; PFCm = medial prefrontal cortex; PFCmp = medial posterior prefrontal cortex; PFCv = ventral prefrontal cortex; SN = Salience Network; Temp = temporal. *p < 0.05, **p < 0.01, ***p < 0.001, ****p < 0.0001. ß = estimate.*
